# Burnout in doctors

**DOI:** 10.1192/j.eurpsy.2025.259

**Published:** 2025-08-26

**Authors:** D. Bhugra

**Affiliations:** Centre for Affective Disorders, Kings College London, London, United Kingdom

## Abstract

**Abstract:**

Burnout is a set of experiences and symptoms which occurred in the context of stress and high ideals in the helping professions who feel drained, exhausted, tired, listless and unable to cope. Burnout leads to disengagement, blunted emotions, helplessness and hopelessness with feeling trapped causing detachment and depression. Prevention of burnout is important and includes interventions at policy, institutional and personal levels. These resources must include financial and human resources where appropriate; prompt and culturally acceptable help is available and easily accessible. Institutions must allocate sufficient funds for well-being of its staff. Various steps can be taken but perhaps most important is accessibility of services in a non-stigmatising manner. These services must be confidential, fit for purpose and widely advertised and accessible. Physical space to rest and access to affordable nutritious food are important. Access to occupational heath services or primary care are important.

Dinesh Bhugra

**Disclosure of Interest:**

None Declared

